# Internet Image Viewer (iiV)

**DOI:** 10.1186/1471-2342-8-10

**Published:** 2008-05-29

**Authors:** Joel T Lee, Kristin R Munch, John V Carlis, José V Pardo

**Affiliations:** 1 Cognitive Neuroimaging Unit, VA Medical Center, Minneapolis, MN 55417, USA; 2 Computer Science Department, University of Minnesota, Minneapolis, MN 55455, USA

## Abstract

**Background:**

Visualizing 3-dimensional (3-D) datasets is an important part of modern neuroimaging research. Many tools address this problem; however, they often fail to address specific needs and flexibility, such as the ability to work with different data formats, to control how and what data are displayed, to interact with values, and to undo mistakes.

**Results:**

iiV, an interactive software program for displaying 3-D brain images, is described. This tool was programmed to solve basic problems in 3-D data visualization. It is written in Java so it is extensible, is platform independent, and can display images within web pages.

iiV displays 3-D images as 2-dimensional (2-D) slices with each slice being an independent object with independent features such as location, zoom, colors, labels, etc. Feature manipulation becomes easier by having a full set of editing capabilities including the following: undo or redo changes; drag, copy, delete and paste objects; and save objects with their features to a file for future editing. It can read multiple standard positron emission tomography (PET) and magnetic resonance imaging (MRI) file formats like ECAT, ECAT7, ANALYZE, NIfTI-1 and DICOM. We present sample applications to illustrate some of the features and capabilities.

**Conclusion:**

iiV is an image display tool with many useful features. It is highly extensible, platform independent, and web-compatible. This report summarizes its features and applications, while illustrating iiV's usefulness to the biomedical imaging community.

## Background

A basic problem in brain research concerns data visualization. The data stored, in a 3- or 4- dimensional (three spatial dimensions and time) image array, become difficult to visualize with standard data graphing or 2-D image display tools. Yet, viewing these data becomes essential for increased understanding, to grasp noise properties, to spot artifacts, and to see relationships in the data that algorithms cannot automatically detect. These issues are not only important for the people performing the research, but also for sharing data and insight with others.

Many tools exist to help in visualizing and presenting data. They range from purely 2-D image displays with no image processing capability to 3-D image displays with special tools for extracting features, statistics, etc. They can be divided into 2-D image manipulation tools, data processing tools, and tools specific to brain imaging. For example, image manipulation tools like Adobe^® ^Photoshop^® ^excel at 2-D pictures and video but do not deal with 3-D data or brain imaging formats. Data processing tools such as MATLAB™, IDL^® ^and the Visualization Toolkit (VTK) [1] have multiple functions for processing and display but require writing programs or scripts for reading, processing, and displaying brain images.

Tools specific to brain imaging include ANALYZE™ [2], SPM [3], AIR [4], MRIcro [5], Brainvox [6], Brain Voyager [7], Stimulate [8], ImageJ [9], Insight Segmentation and Registration Toolkit (ITK) [10], and several others. These tools support, to differing extents, brain imaging data formats, data analysis, and/or display. These programs were primarily designed around a specific processing problem and have display capabilities orientated towards that single problem, often displaying only one data view at a time. ANALYZE and SPM are major packages with multiple processing capabilities but still have display options that are cumbersome and/or inflexible. The display options within these packages do not offer user-friendly features such as rearranging display objects by dragging or undoing accidental changes.

Another notable toolkit is the Medical Imaging Interaction Toolkit (MITK) [11]. This toolkit combines VTK and ITK and adds features that make it easier to interact with these toolkits including multiple consistent views of the same data, interactions and undo/redo. MITK requires writing and compiling programs for specific applications.

Two other Java based image kits include NeatVision [12] and BIL-kit [13]. NeatVision is a medical imaging analysis and software development environment emphasizing computer-aided diagnostics (CAD). The Medical Imaging and Visualization Toolkit (BIL-kit) includes a large range of capabilities from image segmentation to geometric model generation and 3D visualization. Like most toolkits both these require assembling tools to produce specific applications or results. Our simple display application emphasizes visualization and comparison of processed results and does not incorporate extensive processing. Future additions to iiV might look at utilizing features or results from one or both of these toolkits.

Modern brain imaging techniques like positron emission tomography (PET) and magnetic resonance imaging (MRI) generate 3-D arrays of voxels (volume pixels). Voxel values generated by these techniques correspond to measures like radiation decay counts, field intensities, z-values, etc.; these values do not represent visual colors. The third dimension is not easily represented on a flat display screen. Standard 2-D viewers are not sufficient to view these data. The data require techniques to map voxel values to colors, to convert 3-D data into 2-D views, and to discern the areas of the brain under observation relative either to an individual brain or to a standardized brain space.

To visualize counts, field intensities, z-values, etc., these parameters get mapped to a color table where the intensity and/or hue of the colors represent the magnitudes. Visualizing 3-D arrays involves various techniques: displaying sequences of 2-D image slices (one for each of the indices of the 3rd dimension); displaying orthogonal slices; and generating 3-D perspective views.

Data such as MRI scans include enough high resolution anatomical information that brain areas and structures become discernible by experienced viewers. Functional techniques such as PET and functional MRI (fMRI) produce images with relatively little anatomical information and often have to be registered to structural MRI data to reference the underlying anatomy. The functional data may be displayed side-by-side with the anatomical MRI or overlaid on top of the MRI. When overlaying, the non-significant portions of the functional data can be omitted (completely transparent), so that portions of the underlying structural MRI can be seen. Alternatively, the functional data can have slight transparency. Or, the functional data can provide color hues while the MRI provides grey level intensities.

Data from all brain imaging methods often get mapped to a standard space to allow comparison across subjects, studies, and sites. Many standard spaces exist as well as multiple methods for registering to standard spaces. The two most common brain spaces are the Talairach space [14] and the MNI space [15,16]. Registration techniques are found in programs such as SPM, AIR, Automated PET Activation Analysis Package [17-20] and Neurostat [21]. Techniques range from simple linear scaling to complex nonlinear warping.

An important part of data display is user interaction. Allowing the user to interactively modify features of displayed data can help them grasp properties of the data to a greater extent than when viewing static displays. Even interactive editing features like undo/redo can allow a user to flip back and forth between changes, assisting in the identification of noteworthy differences.

A last background note indicates the variety of brain imaging data formats and the problem of reading these formats. Every camera and software tool seems to produce a different file format. Some common PET formats include Siemens/CTI ECAT 6 and 7, ANALYZE, NIfTI [22], and DICOM^®^. Most cameras are adopting the DICOM standard, but this standard incorporates many variations – even if a tool can read some DICOM files, it may require modifications to read data from a new DICOM source. Although data storage is getting cheaper, it is helpful if a tool supports a given format so that converted files do not have to be created, tracked and stored outside of the tool.

A relatively new format is NIfTI. NIfTI extends the ANALYZE 7.5 format incorporating increased flexibility, and storage of transform parameters to map voxel locations to standardized space. NIfTI specifies that compliant tools incorporate this mapping to indicate correct orientations. When iiV reads NIfTI formatted files it automatically displays the data with the most complete transform mapping available in the header.

As noted above, many existing tools address the visualization problem but do not have the needed flexibility for all research groups, which leads many researchers to develop their own display tools. This paper introduces a new tool called iiV (internet image Viewer), developed by the Cognitive Neuroimaging Unit, as our own display tool. Like other tools, it does not address all problems, but we feel it has reached a high enough level of utility to be useful to other groups. In the following sections we describe iiV's implementation, discuss its major features, show examples of various uses, as well as present future directions.

## Implementation

The following outlines some of iiV's details concerning the software design and implementation.

### A. Programming environment

iiV is written in Java 1.1 and utilizes Swing, BeanShell 2.0b and ACME Lab GIF Encoder packages. iiV makes strong use of Java's object oriented paradigm, multithreading, and object reflection. iiV's display area and user interface build directly upon Swing's component oriented, single-threaded programming model, and model-view-controller (MVC) graphical user interface (GUI) framework. BeanShell provides iiV with a scripting environment that emulates the Java language syntax. iiV utilizes this scripting for initialization, for saving displayed information, for creation of animated presentations, and for cut and paste abilities. ACME Lab tools are used for GIF image encoding of the display area.

#### 1) Java

Object oriented programming becomes incorporated with Java's structure of classes that define contracts with other code in the form of methods. Object oriented programming separates the notion of what is to be done, defined by the semantics of class (object) methods, from how it is done, which is defined by the method's code. This separation of "what" and "how" helps break up programming tasks into units that are easier to manage and debug. Object oriented programming enables overloading, allowing different methods to have the same name but different semantics to perform similar tasks; inheritance, allowing classes to extend the utility of previously defined classes; and interfaces, defining methods but not code (classes implementing the interface must provide the code).

Multithreading allows two or more sections of the same program to run simultaneously. Unlike separate programs, the threads share memory and resources. Java and the underlying operating system handle the distribution of CPU time to the different threads much like to different programs. Multithreading allows time consuming tasks like file input/output (I/O) to run simultaneously with tasks like GUI interaction that need to be responsive. Neither of these types of tasks tends to be CPU intensive, but if the GUI had to wait for I/O, the user might have to wait as well. Also, other tasks can be performed while waiting. Processing some tasks like I/O in another thread also gives the possibility of interrupting that thread if it takes too long.

Since multiple threads of the same program share resources, there needs to be a mechanism to prevent one thread from accessing a resource while it is invalid or not ready because another thread is modifying it. Java provides an object based synchronization lock that only one thread can grab at a time. Other threads requesting the lock freeze until the prior thread releases the lock. Synchronization locking can lead to deadlocks with two threads waiting on each other to release separate locks. Synchronization deadlocks can be intermittent and hard to debug. iiV avoids synchronization problems by only using synchronization around local code with limited branching, e.g., synchronizing only around local private functions or simple functions (like system math calls) that should not involve further synchronization.

The Java Reflection application programmer's interface (API) gives the programmer tools to create instances of, and call methods for, classes at run time without knowledge of the class at the compile time. iiV uses reflection to add dialogs, new display components types, and file types. This allows users to plug in new dialogs and file types at run time. It also allows iiV to run without loading unnecessary dialogs and file types – that can save time when running across the Internet within a web browser.

#### 2) Swing

**Figure 1:**
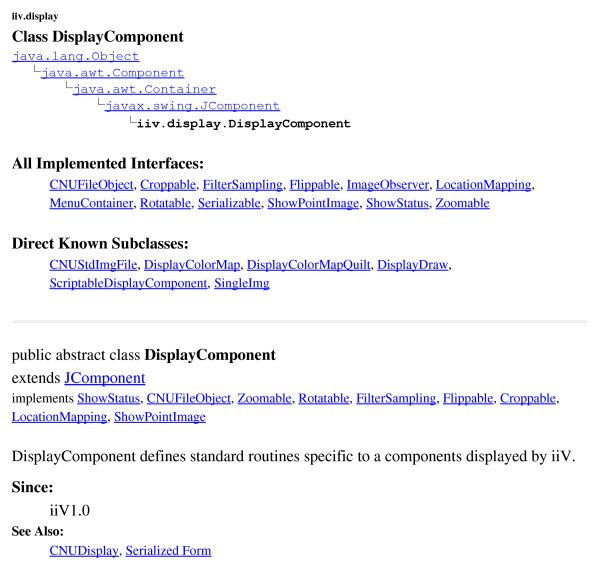
iiv.display. DisplayComponent class structure from iiV API documentation.

**Figure 2:**
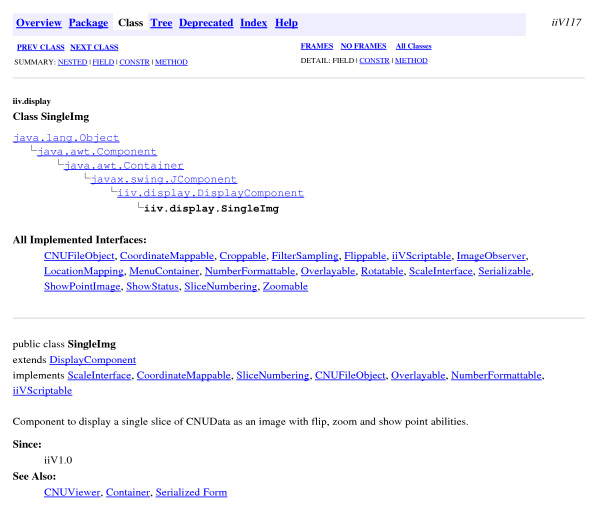
iiv.display. SingleImg class structure from iiV API documentation.

**Figure 3:**
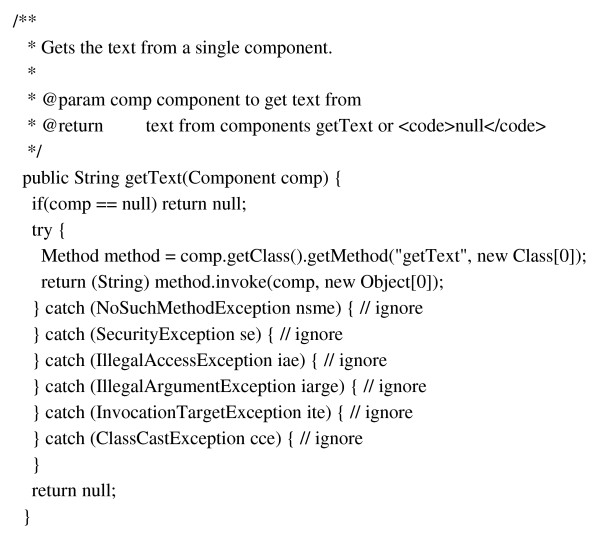
Sample code demonstrating how java reflection is used to get a components feature.

iiV utilizes the Swing GUI package which incorporates a component-oriented, single-threaded programming model, and model-view-controller GUI framework. The component oriented notion of Swing defines each component of the GUI as a class object. iiV not only incorporates the component oriented framework for its GUI, but also for its display area. The objects that iiV is able to display are subclasses of java.awt.Component. Most are subclasses of iiv.display.DisplayComponent, as seen in Fig. 1, which shows part of its API documentation. The iiv.display.DisplayComponent class implements most of the interfaces iiV uses for controlling displayed object features like Croppable, Flippable, and Zoomable. The iiV.display.SingleImg class, shown in Fig. 2, is the primary class for displaying single slices from 4-D data files. It implements some additional interfaces such as CoordinateMappable, Overlayable, and ScaleInterface. Display component features are also accessed via the Java reflection package as highlighted in the code sample in Fig. 3.

GUI interaction requires a lot of synchronization. An early version of iiV utilized the Java's Abstract Windowing Toolkit (AWT) directly and was prone to synchronization deadlocks. With the introduction of Java's Swing components, the probability of deadlocks was reduced by performing all GUI updates within a single Swing event thread. A section of code that needs to interact with the GUI inserts code as a runnable object in the queue for the Swing event thread. The Swing event thread executes objects from this queue one after another. iiV is careful not insert code into the queue that would consume a lot of processing time or introduce new synchronization locks.

In the framework of the model-view-controller, the model represents data; the view renders the data as a user interface element; and the controller responds to events that produce updates to the model and/or view. In iiV this framework defines a single model with associated action/event(s) which can associate with multiple GUI components displayed in different menus and/or containers. iiV also extends this framework to track mouse events over an image and displays data values, crosshairs, and updated displays of brain slices showing related data.

#### 3) BeanShell

iiV utilizes BeanShell for scripting. BeanShell allows a script to have full access to the power of java. Since scripts are basic text files, they can be edited with any text editor. Scripts are used in the following ways in iiV:

• Scripts are used for saving iiV settings to bring iiV up in the same mode later.

• Scripts are used for saving what is displayed so it can be redisplayed later.

• Scripts can be used for automating display sequences such as showing slices sequentially.

• Cut and paste utilizes scripting. This avoids problems with serialization, maintains information in human readable form, and allows pasting into other copies of iiV as well as text editors.

#### 4) ACME Labs GIF encoder

This is a java tool used to encode the display as a GIF formatted image file.

### B. iiV structure

**Figure 4:**
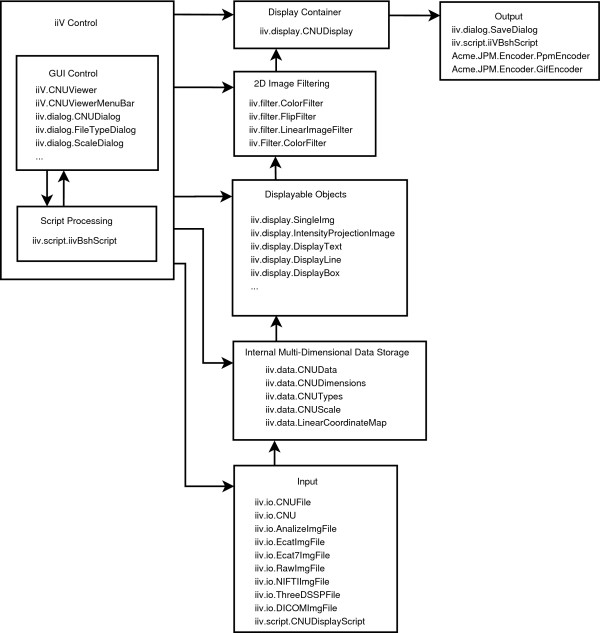
**This basic iiV architecture diagram shows the control and flow of data from input files to display and saving.** Each block lists some of the primary Java classes involved.

iiV is divided into 9 different packages, the top level iiv package and 8 sub-packages: iiv.display, iiv.data, iiv.dialog, iiv.io, iiv.gui, iiv.filter, iiv.script, and iiv.util. A short overview of each package and how they interact is listed below. Fig. 4 shows the basic architecture emphasizing how data flows from files to display, and the primary Java classes involved. The total API is available on line [23]. It includes a complete list of all classes and interfaces for each package as well as their inheritance structure and summaries of their public fields, constructors, and methods.

1. The top level package iiv contains the main class CNUViewer that extends the java.awt.Applet. Extending the Applet class allows iiV to run inside a web browser. CNUViewer controls setup, default settings, reading and displaying data, access to display features, and access to other GUI's in the iiv.dialog package. The iiv package also includes two other classes, CNUViewerActions and CNUViewerMenubar, that define gui access to main features of the CNUViewer and iiv.display.CNUDisplay classes.

2. The iiv.display package contains the main display container class, displayable classes, and feature control interfaces. The class CNUDisplay is the main controller and container for displaying objects. The primary displayable object is the SingleImg class which displays slices from iiv.data.CNUData objects. Properties from current display settings and the data itself determine default display properties of the slices. These properties can be modified by display area GUI interaction as well as dialog windows and scripts. Most changes to the display area and objects go through special display functions to track changes and allow for undo/redo via the iiv.util.UndoRedo class.

3. The iiv.data package includes classes for storing, scaling, and coordinate mapping of data. These classes help with storing and accessing data including access to multi-dimensional arrays of different data types, conversions between data types, scaling of data, and mapping coordinates. The CNUData class falls under this package and is the main class for storing data displayed by the iiv.display.SingleImg class.

4. The iiv.dialog package has dialog classes that define windows for controlling and interacting with displayed items. They talk through the main controller (iiv.CNUViewer), display area (iiv.display.CNUDisplay) and displayed objects API to control how and what is displayed.

5. The iiv.io package contains file access classes including the different file type interpreters. Most of these classes extend iiv.data.CNUData class which is displayable by the iiv.display.SingleImg class or the iiv.display.IntensityProjectionImage class.

6. The iiv.gui package defines some GUI components that extend some of the standard Swing components. The class CNURowLayoutManager is in this package and is used by the main display container, iiv.display.CNUDisplay, to organize displayed objects in rows.

7. The iiv.filter package defines filter classes used to zoom, rotate, crop, etc. images. Most of these get inserted between raw 2-D data and the actual displayed image inside the iiv.display.SingleImg class.

8. The iiv.script package defines classes for dealing with scripts. The class iiVBshScript falls in this package and is the main class for running Bean Shell scripts.

9. The iiv.util package contains some general utility classes including the UndoRedo class for undo/redo capabilities.

### C. Undo/redo

To allow undo/redo, iiV has an undo/redo controller class, iiv.util.UndoRedo, that modifiers of displayed objects register commands with – one command to undo and one to redo each modification. These are stored as iiv.util.DoCommands objects that store objects, methods, and method arguments. Because of the stored object and argument objects, undo/redo history becomes searchable for file objects, which reduces the need to read files multiple times.

### D. Speed and memory considerations

Memory and speed have not been major concerns of iiV's design and methodology. Speed is not a major concern because iiV is mostly GUI, and today's processors handle this easily. Memory is more of a problem because 3-D image files can be large, and undo/redo can maintain a large history of unused objects in memory.

The Java Virtual Machine (JVM), when invoked, specifies maximum memory allocations. With today's computers, the JVM can be invoked with large amounts of memory. Java has a good garbage collector that removes objects when they are no longer referenced.

iiV loads file data completely into memory and maintains it there as long as it is referenced by any object enabling fast access to the original data. This includes objects referenced by undo/redo history that may no longer be displayed. To reduce waste, iiV searches existing objects before loading new files and allows clearing and/or turning off undo/redo history.

When run as an applet, load times across the web become the major concern. Load times involve both the size of iiV jar files (iiV executable code) and data files. To help with this, iiV, as noted above, can run without all its dialogs and file types. Also, data files can be compressed with GNU zip (gzip).

### E. Neuroimaging data display

The primary technique chosen by iiV for viewing 3-D brain data is a combination of 2-D slices. Slice viewing remains the most complete and clean way to view brain data. Currently, only slices orthogonal to the major axes are available. They are simplest to generate and do not introduce new distortions. To get a perspective of the whole brain, all slices ranging from one side of the brain to the other (left to right, front to back, or top to bottom) can be displayed. To be more concise, but with reduced information, three orthogonal slices may be displayed. The underlying goal is to provide the right combination of slices with proper orientations to help visualize positions of, and relationships between, areas of interest. This is why iiV supports a very flexible display area with image objects that can be positioned anywhere, including overlapping slices or other objects.

To relate functional data to anatomical data, the anatomy can be displayed as slice objects next to or behind related functional slices. When the anatomical data are located in the background, parts of the functional data may be set to be transparent – usually where voxels have small or insignificant values. This overlapping requires the functional and anatomical data sets be co-registered. Keeping the anatomical and functional data as separate objects allows independent modification of colors, contrast, thresholds, etc. iiV allows grouping to lock related objects together.

A second viewing technique available in iiV is intensity projections. This technique produces a seemingly transparent or x-ray view of the whole brain as a single 2-D view. Each voxel in the 2-D view shows the maximum (or minimum) intensity voxel selected from all voxels along a line parallel to a major axis. Intensity projections may be done parallel to any of the 3 major axes.

A third viewing technique is available for 3-D stereotactic surface projections (3D-SSP) data. This is data generated outside of iiV utilizing the NEUROSTAT [21] software package. The data is stored as a list of indices and voxel values for data projected to the surface of a brain. iiV reads and keeps track of these indices while displaying 8 surface views of the voxel values – left hemisphere, right hemisphere, anterior, posterior, superior, inferior, left medial hemisphere and right medial hemisphere. The 3D-SSP data is already in a standardized space, and iiV includes a spatial mapping to track Talairach coordinates for all locations on the surface views.

iiV does not include the ability to generate its own 3-D perspective views (beyond intensity projections) or to perform the actual registration of data to standard spaces. These problems depend greatly on the particular data type and often become computationally intensive, so they are best left to specialized tools. iiV can display 3-D perspective views generated by other software, and can also display coordinates in standard space when given linear mapping constants (or for a more complex mapping, given a java object that implements iiV's coordinate mapping interface).

## Results and discussion

To address limitations of existing tools, our laboratory developed its own internal visualization tool, iiV (internet image Viewer). We were able within iiV to address many of the problems noted in the background section above, including the ability to control the types of data for display, to control how data are displayed, and to add many interactive capabilities to the display itself. In particular, iiV deals with data voxels to color representation, slice viewing, overlaying, mapping locations to standard space, and visualizing any number of slices simultaneously from various perspectives. The types of data we can display have grown as we have encountered new formats from different machines and collaborators. Our basic application programmer's interface (API) allows easy addition of new formats.

**Figure 5:**
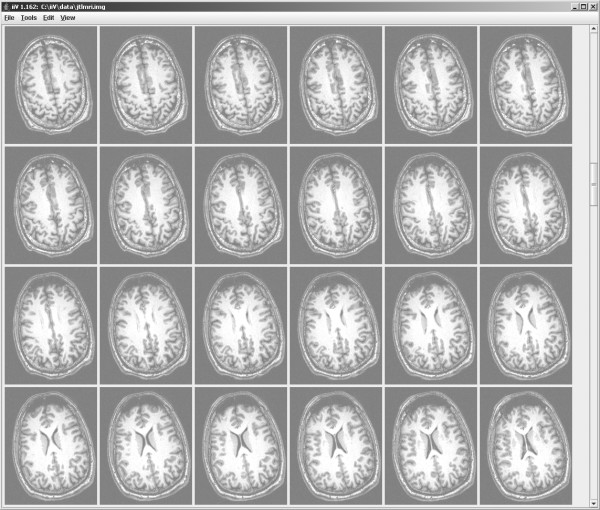
**Default display of an MRI stored as signed short.** All transverse slices are displayed by default with the full possible short (16-bit) data range mapped to a 256 grey level color map. The maximum value of 32,767 maps to white and the minimum value -32,768 maps to black. Zero maps to the mid grey value which is why the background areas on the slices are grey not black. The MRI actually contained no values less than zero and contrast could be improved by scaling to show positive values only with zero mapped to black as in Fig. 6. By default there are six columns and only 24 of 255 transverse slices fit on the computer display, the rest could be scrolled to for display.

iiV is a full-featured tool for displaying brain imaging data. The primary display objects are 2-D slices constructed after reading a 3-D data file. Fig. 5 shows transverse slices of an MRI displayed within iiV with all the default settings. By default, upon reading a data file, iiV displays all transverse slices mapping the full voxel range to 256 grey level colors. Each displayed slice is an object with independent features that can be manipulated.

iiV has grown in utility and flexibility to the point of including many user friendly interactive features such as object dragging, copy, delete, paste, undo/redo, and a script language. Additional interactive capabilities include the ability to change object features such as zoom, cropping, rotation, scaling, thresholds, and color tables. Also, iiV allows selecting a voxel to view the original voxel value, quantified voxel value, location indices of the voxel, and location mapped to a standard coordinate space. Another interactive ability allows selecting a voxel and having slices from different views or other data automatically update to highlight the same or related voxel location.

The following lists the major features of iiV:

• Provides flexibility, portability, and user-friendliness.

• Performs the major utilities for displaying brain imaging data for improved understanding, sharing, and publishing.

• Displays 3-D data as slice objects orthogonal to the major axes.

• Displays 3-D data as maximum or minimum intensity projections parallel to the major axes.

• Displays 3-D stereotactic surface projections with location mapping to Talairach space.

• Presents multiple slices from the same or different files with each slice having independent features.

• Allows user to organize varying views of data for comparison.

• Allows user to select a location in one object, then to automatically highlight the related location in other objects with crosshairs and slice tracking.

• Translates locations to standard spaces such as that of Talairach.

• Provides standard edit features including copy, delete, paste, and undo/redo.

• Uses Java for cross-platform compatibility.

• Interacts easily with other Java applications.

• Runs optionally as an applet within web pages or embedded within other Java applications.

The following sections expound on these features.

### A. Viewing slices

**Figure 6:**
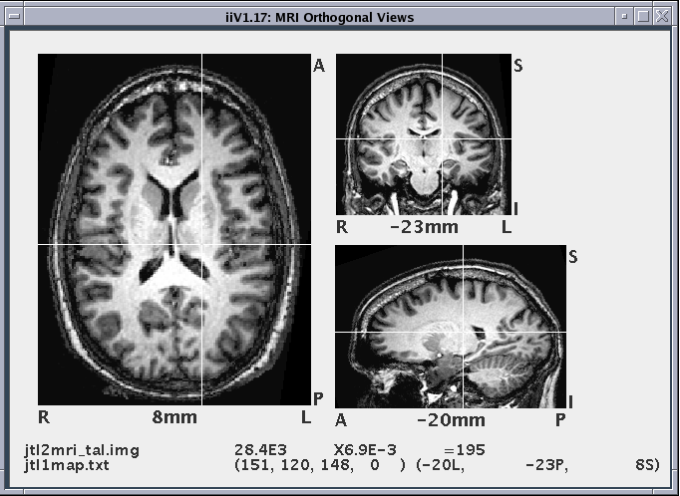
**This shows orthogonal views of an MRI with a transverse view on the left, coronal view on top right, and sagittal view on bottom right.** The transverse view is zoomed 1.5 times as large as the others. White crosshairs call out the same voxel in each view. A show point display line object at the bottom displays voxel information for the crosshair value both as a raw value from the file and quantified. The show point display line also shows the data file name, quantification factor, coordinate mapping file name, index to the voxel and mapped location of the voxel. In an active iiV display, the views and show point display line would automatically update to show a selected voxel.

Data from 3-D files are displayed as slices orthogonal to any of the three major axes (see Fig. 6). When displaying brain images, slices are considered transverse (parallel slices orthogonal to the z-axis running from the bottom to the top of the brain), coronal (parallel slices orthogonal to the y-axis running from the back to the front of the brain) or sagittal (parallel slices orthogonal to the x-axis running from the left to the right of the brain). This paper uses the term slice location to refer to the z-axis location for transverse slices, the y-axis location for coronal slices, and the x-axis location for sagittal slices.

**Figure 7:**
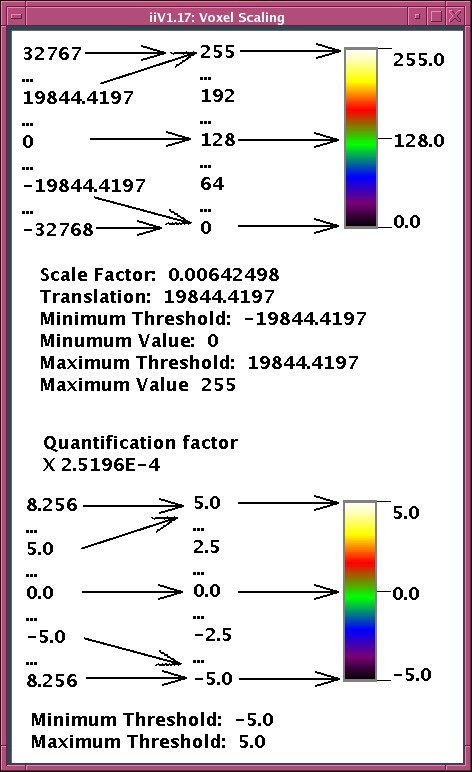
**The top picture outlines mapping short words (16 bit) whose values can range from -32768 to +32767 to color values.** This is done with linear scale factors (middle table) that include thresholds mapping values that would fall outside the color range to the ends. Because data words often include a quantification factor, the scaling can also be viewed in terms of quantified values (bottom picture) which may be more user-friendly by having units representing parameters such as t-values.

iiV maintains the original 3-D data in memory to allow quick updating of scaling, colors, and slice locations as well as remapping the display back to voxel values. Voxels are mapped to display colors via linear scaling (with possible maximum and minimum thresholds) and a color look-up table with 256 values (see Fig. 7). Scaling and color tables can be different for each slice displayed. Scale thresholds allow hiding voxels below or above a value. For example, when viewing normalized PET radiation count data, an investigator may not wish to see values below some minimum count corresponding to background noise. Color tables can include transparent colors that allow see-through to objects in the background.

Displayed slices can be individually moved, zoomed, rotated, flipped, cropped, copied, and deleted. They may also employ new voxel scale factors, color maps, and coordinate maps. Slice locations can be incremented or decremented to display any slice parallel to the initial slice. Automatic annotation shows labels for orientation and slice location. Fig. 6 shows slices annotated with the following labels: R, for right; L, for left; A, for anterior; P, for posterior; I, for inferior; and S, for superior. The number at the center bottom of each slice specifies the slice location in millimeters in Talairach coordinates. The annotation permits control of color, font and number format.

For examining specific voxels in the brain, iiV shows voxel values for locations selected with the mouse over an image. The values are displayed in a separate dialog window or in a show point display line object as seen at the bottom of Fig. 6. Each show point display line is actually two lines. The first line shows the original data file name, the raw voxel value, the quantification factor, and the quantified voxel value. The second line shows the mapping name, the raw location indices, and the location mapped into a standard space. A show point display line may show values for the data the mouse is currently selecting, or it may be keyed to show values for specific data. For example, the mouse may select a voxel over an MRI, and the show point display line shows the corresponding voxel from PET data. There may be multiple show point display lines allowing the user to view corresponding voxel values from multiple sources.

To help locate specific voxels over multiple displayed slices, iiV includes the ability to display crosshairs, and to automatically update crosshair locations or slice locations. The white lines in Fig. 6 demonstrate how crosshairs highlight a voxel. The crosshairs can automatically update to show the same location as that selected by the mouse over any image. If the selected location is in a different slice then either the crosshairs become dashed lines or, with automatic slice updating, the slice location updates as well as the crosshairs. This automatic updating feature works well when viewing orthogonal slices of the same data. For example, selecting a voxel in the transverse view can automatically update the sagittal and coronal views to display the slices containing the selected voxel highlighted with crosshairs. Additionally, a voxel selected from one data set can reveal the corresponding voxel from another data set including data from a different subject or modality. If the data is not in the same space, iiV calculates the corresponding locations via coordinate mapping transforms.

As mentioned previously, voxels can be mapped to transparent colors. This allows overlaying slices on top of each other making voxels from the underlaid slice visible through the transparent voxels. To facilitate overlaying, iiV allows data with the same dimensions to be automatically overlaid. This auto-overlay process places a top slice over a displayed bottom slice while duplicating the view mode (transverse, sagittal or coronal), slice number, zoom and cropping. For data in the same space, this ensures the overlay slice displays spatially related data at the same locations as the slice behind it. An example of overlaying would be viewing a subject's PET data superimposed upon the subject's structural MRI; where the PET counts are below a specified minimum, the MRI is visible instead of the PET. Another example is shown in the bottom right of Fig. 10 where t-values are shown overlaid on top of a standard MRI image.

### B. Displaying intensity projections

**Figure 8:**
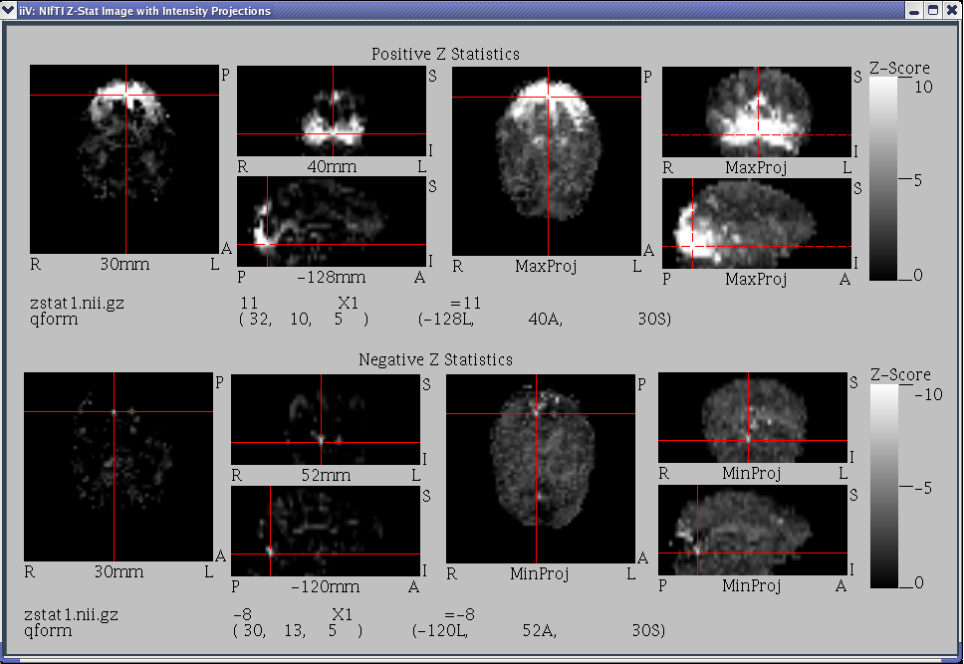
**This figure contains images generated from a NIfTI Z-Stat sample data file downloaded from the NIfTI web site [22].** The top half shows positive Z values with the 3 orthogonal images on the left representing the standard slices and the 3 orthogonal images on the right representing maximum intensity projections. At the bottom of the top half is a show point line displaying the selected voxel location and value. The bottom half shows negative Z values, minimum intensity projections, and the selected voxel location and value for the bottom half. Notice the dashed crosshairs over the right most maximum intensity projections indicates that the maximum intensity along the line perpendicular to each of those views did not occur at the currently selected voxel (the current selection was made over the transverse maximum projection).

Data from 3-D files are also viewable as intensity projection images (see Fig. 8). As with standard slices, intensity projections appear as 2-D images orthogonal to one of the three major axes – except each displayed voxel is the maximum (or minimum) value from all voxels along a line parallel to the axis. A projection can be restricted to a range of orthogonal indices allowing for half brain projections, etc.

Most features of standard slices are available except those related to the orthogonal dimension. Automatic labeling shows orientation values but not orthogonal location (slice location for normal slices), because it may vary from voxel to voxel within the projected view. When voxels are selected over projected views, iiV uses the original indices of the projected voxel for displaying and coordinate mapping. When automatically updating crosshairs over projected views the crosshairs become dashed lines when a voxel in another view is selected that has different indices from the original projected voxel. Conversely, when a voxel over a projection view is selected, standard slice views, with automatic crosshair and/or slice tracking enabled, update to show location of the original projected voxel.

### C. Displaying 3-D stereotactic surface projections

**Figure 9:**
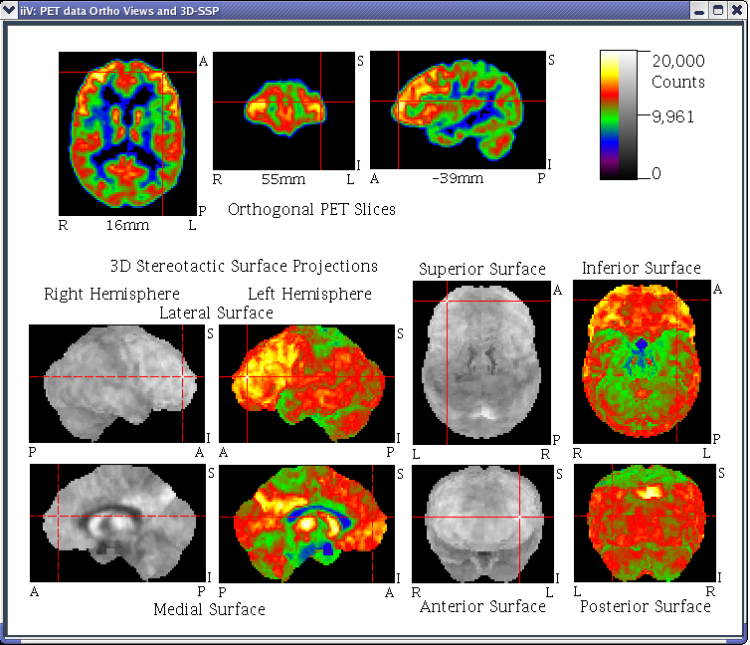
**The top 3 images show orthogonal views of raw PET data.** The bottom 8 images show the same data converted to 3D stereotactic surface projections by NEUROSTAT software [20]. The surfaces are shown with both gray and pseudo color tables to highlight the influence of color on contrast. Solid crosshairs indicate an image is showing the currently selected voxel, where as dashed crosshairs indicate the currently selected voxel is not visible in the image but is located somewhere orthogonal to the crosshairs. Notice the left hemisphere lateral surface, the superior surface, the anterior surface and the orthogonal slices are all showing the currently selected voxel while the other surface views are not.

Three-D stereotactic surface projections (3D-SSP) data, generated by the NEUROSTAT software package, are viewable as 8 surface views – left hemisphere, right hemisphere, anterior, posterior, superior, inferior, left medial hemisphere and right medial hemisphere (See Fig. 9). The data is read in from a list of indices with values and converted to a 3-D data with dimensions 128 × 128 × 8, where the 3^rd ^dimension corresponds to the 8 surface views. The data is viewed as standard slices with a special default coordinate map for each slice. This coordinate mapping allows tracking related points with slices of standard 3-D image files.

### D. Displaying other objects

In addition to viewing data, iiV has other built-in display objects, mainly for annotation. These include text, simple shape objects, color bars, and show point display lines. The simple shapes include lines with or without arrow heads, boxes and spheres (see Fig. 7 for text, color bar, line and box examples – boxes outline the color bars). Show point display lines track and display voxel locations and values selected over an image (the bottom of Fig. 6 contains a show point display line). These objects can be zoomed, cropped, flipped and rotated just like image slices. Object color, font, and number formatting can also be controlled.

There are three other built-in objects that can be displayed in the display area but are not normal display objects. These are a location cursor, grid lines, and paper outline. The location cursor indicates where the next object added will appear. This location can be dragged via mouse input and is automatically updated when new objects are added. The grid lines are equally spaced horizontal and vertical lines that can have any size spacing or any color and act as a reference for aligning objects. The dialog box for grid control allows snapping objects to align precisely with grid lines. The paper outline is a box that can be calibrated to correspond to what will fit when printing to paper.

### E. User interface

The design of the iiV user interface tries to maximize display area while maintaining utility and flexibility. User friendliness is another high priority, but not at the sacrifice of functionality.

iiV maximizes its display area by keeping control functions in menus or dialog windows that are usually hidden. Fig. 5 shows the standard display window. If objects in the display area do not fit within the window, scrollbars appear along the right and/or lower edge of the display area. Scrollbars create a virtual display area as large as memory allows. The menu bar at the top of the display can be hidden to further maximize the visible area.

iiV's interface utility and flexibility comes from the ability to place any type of display object anywhere in the display area and to individually control object features. Objects are initially added to the display area without overlap, left to right, and top to bottom with a set number per row. Once displayed the user can drag objects anywhere within the display. Feature control is done with pull down menus or in specialized dialog windows. New object types and feature control dialogs can be added to iiV without recompiling as long as they extend the correct Java class.

For ease of use, many of iiV's edit and mouse functions mimic standard editors. Examples include selecting objects by left clicking on them or right clicking to pop up a menu with edit features such as copy, delete, and paste.

### F. Editing and scripting

To enhance user-friendliness, iiV includes many features that standard editors offer. These include copy, delete, paste, and undo/redo. Also, since iiV works with objects that may have set relationships to each other, the program allows for grouping and ungrouping. iiV includes the ability to print or save the display as a standard 2-D image. Furthermore, the display can be saved as a script.

Scripting is a valuable feature that greatly extends iiV's flexibility. It allows redisplay in future sessions restoring full editing and interactive abilities. Since the script is stored as a text file, viewing different data with the same complex layout simply involves text editing and replacing file names with those for the alternate data. In this way a script can act as a template. iiV automatically runs the script name ".cnu" if it exists in the user's home directory which allows setting individual preferences.

Since iiV processes scripts with BeanShell, iiV scripts have full access to the Java programming environment and can perform many complex tasks such as running interactive animations or creating complex displays with prompting for file names.

### G. Run environments

iiV is run within a Java Virtual Machine (JVM). The availability of JVM for most major operating systems makes it extremely portable.

iiV can run as an applet within a web browser. This is convenient for interactive display of brain imaging data within web sites. As an applet, iiV has almost all the utility of a stand-alone version. Browsers put security restriction on Java applets causing problems with some features and with some scripts. These restrictions may range from severe (e.g., applets unable to run at all) to lenient (e.g., treating applets as local code). One common restriction is that applets are not allowed to save to the local disk. Applets also cannot copy and paste to or from the system clipboard. Some restrictions can be overcome by changing applet security restrictions within the browser. There are also procedures to run certain applets as "trusted" with fewer restrictions than other applets. Even with most common applet restrictions, iiV is a valuable interactive web display tool.

For simple data presentations and to decrease upload times to web browsers, iiV can run without loading any dialogs, menus or file formats. To this end, dialogs, menus and file formats are not compiled into iiV but are referenced by name and loaded when required utilizing the Java reflection API. This strategy allows iiV to load and run without the availability of certain tools. A core set of normally referenced classes must exist to prevent Java from aborting with class loading exceptions.

**Figure 10:**
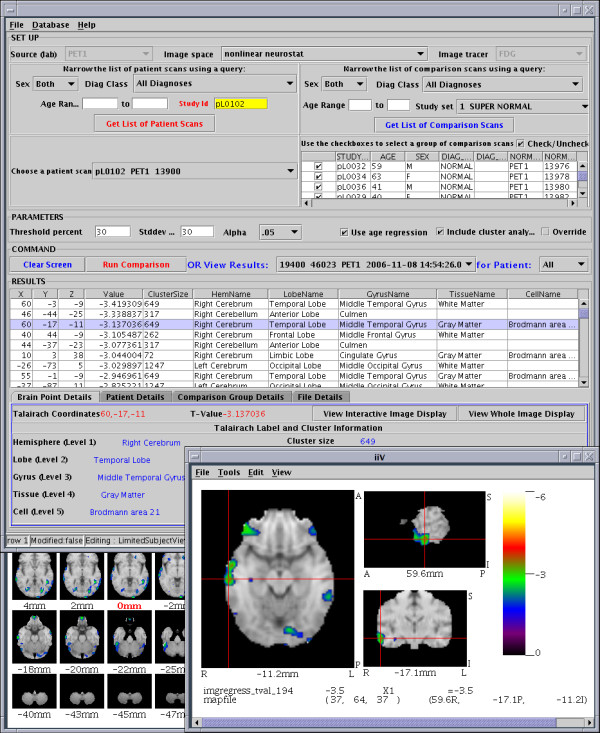
**Oracle database interface tool that automates comparisons between a single subject to a group.** The top half of the interface selects the subject, comparison group, and parameters. Results are shown as a list of peak t-values with Talairach locations and labels from the Talairach Daemon located near the middle. iiV interacts with the tool displaying a selected location (highlighted on the results list) in orthogonal views (lower right). Selecting a different result line causes iiV to update and inversely clicking a location over iiV selects the nearest peak in the results list. The tool can also pop up another copy of iiV showing results over all transverse slices (partially visible lower left). Images shown in this figure were warped to a standard space using the Automated PET Activation Analysis Package [17-20] and overlaid on a reference MRI that came with that package.

iiV can easily be invoked and controlled from other software tools, especially those written in or with simple interfaces to Java. For example, our database management system (DBMS; Oracle v9i, Cupertino, CA) [24] can compare a patient's scan to a normative dataset. It can invoke iiV to display the results and to update crosshairs and slice locations upon user selection of specific results from a table (see Fig. 10). Selecting a point within iiV does the inverse – highlighting the nearest specific result in the table.

### H. Extending iiV

iiV is easily extended in multiple ways:

• New file types can be added that extend the iiv.io.CNUImgFile class. iiV doesn't have to be modified or recompiled to recognize the new class. The new class name is given to iiV either in the file type dialog or via a script command. After adding a new class name, the class name appears in the pull down menu for file types. Script commands can run every time iiV becomes invoked to always have the new file type available.

• iiV may also be extended with display objects that do not adhere to the standard 4-D iiv.io.CNUImgFile class. These objects need only extend the java.awt.Component class. They should also implement the iiv.script.scriptable interface in order to be saved as a script. They may implement interfaces such as iiv.script.iiVScriptable; iiv.filter.Zoomable; or iiv.filter.Croppable for additional utility.

• New control dialogs that extend java.awt.Dialog or iiv.dialog.CNUDialog can be added for functionality. These dialogs are readily invoked via script commands and appear in the dialog view menu.

Many other extensions to iiV are possible since it is written in Java and has clean class structure.

### I. Examples of usage

iiV has proven effective; it is extensively used in our laboratory. The following list highlights sample uses of iiV:

• Viewing raw PET data – our first application of iiV was to display raw PET data immediately after reconstruction to look for basic problems such as abnormally low counts or head motion.

• Exploring PET comparison results – iiV is our primary tool for displaying voxelwise statistical maps of processed PET data.

• Creating presentations and journal figures – iiV is our main tool for creating data visualizations to share with others through presentations and publications.

• Viewing the results of database queries – we are currently developing Oracle database tools that work with a database of PET images to perform statistical analysis based on query parameters selected by the user. These queries produce t-value images as well as lists of significant t-value peaks with their Talairach locations and probable brain regions. The database interaction tool is written in Java and easily interfaces with iiV to display the locations as the user selects them from the list. iiV displays the locations as crosshairs over orthogonal slices of the t-value image overlaid on a reference MRI. This gives the user a better grasp of the location as well as the size and extent of the activation region surrounding a peak location (Fig. 10).

• Displaying within web pages – the next step in the development of database tools includes an interface for users to perform queries via a web browser to have iiV display results within the browser. We currently have a demonstration of iiV that runs within a web browser available through the iiV home page [25].

### J. Future direction

iiV continues to be a work in progress. One primary incentive has been the flexibility of having our own in-house tool which allows adding features as needs arise. Some features that we would like to add in the near future include the following:

• Re-sliced views – we plan to enable viewing slices at arbitrary angles not just perpendicular to the major axes. This requires re-slicing the data while maintaining fast inverse mapping to the original data for voxel location and display of associated parameter values.

• 3-D views – with 3-D packages available in Java, it will soon be easy to generate 3-D perspective views within iiV. Although other packages can generate 3-D views outside of iiV, implementing 3-D views would enable immediate access to voxel location and parameter values. Currently the only 3-D views available in iiV are 3D-SSP views generated by NEUROSTAT [21] and intensity projections.

• Display results of the Talairach daemon – we plan to have iiV describe brain locations based on voxel locations mapped to Talairch locations as referenced by the Tairach Daemon [26].

• Display results of the MNI probabilistic atlas – we plan to have iiV describe probabilistic brain locations mapped to the MNI template as referenced by the MNI atlas [27].

• Object inspection dialog – an object inspection dialog would enable the inclusion of features of unknown displayed objects and associated parameters. This would ease the addition of new object types.

## Conclusion

Data visualization is a problem facing brain researchers on a daily basis, therefore promoting the development of new tools. No tool has the flexibility required by all researchers. Our in-house tool has reached a utilitarian level making it valuable to many researchers. iiV is primarily good at displaying slices from brain data in a flexible environment for object manipulation with robust editing features such as copy, paste, undo/redo, group/ungroup. iiV provides useful feedback to the user including the display of voxel location and parameter values; crosshair tracking; automatic slice updating; and overlay features. iiV is written in Java and will run under all major operating systems. iiV is a proven multipurpose tool that we plan to continue developing well into the future. This report provides an introduction to iiV's many features to promote wider use in biomedical imaging.

## Availability and requirements

• Project name: iiV

• Project home page: 

• Operating system(s): Java Virtual Machine (available for all major operating systems)

• Programming language: Java

• Other requirements: BeanShell available at .

• License: none

• Any restrictions to use by non-academics: none

## Abbreviations

API: application programmer's interface; AWT: Abstract Windowing Toolkit; BIL-kit: Medical Imaging and Visualization Toolkit; CAD: computer-aided diagnostics; DBMS: database management system; fMRI: functional MRI; GUI: graphical user interface; iiV: internet image Viewer; I/O: input/output; ITK: Insight Segmentation and Registration Toolkit; JVM: Java Virtual Machine; MITK: Medical Imaging Interaction Toolkit; MRI: magnetic resonance imaging; MVC: model-view-controller; PET: positron emission tomography; voxel: volume pixel; VTK: Visualization Toolkit; 2-D: 2-dimensional; 3-D: 3-dimensional; 3D-SSP: 3-D stereotactic surface projection.

## Competing interests

The authors declare that they have no competing interests.

## Authors' contributions

JTL designed and programmed the software and wrote major portions the paper. JVP oversaw writing the software and wrote and edited portions the paper. KRM contributed to portions of the software including application to database results and wrote and edited portions the paper. JVC helped to conceive portions of the software and helped editing the paper. All authors read and approved the final paper.

## Pre-publication history

The pre-publication history for this paper can be accessed here:


